# Neoadjuvant Endocrine Therapy in Breast Cancer: Current Knowledge and Future Perspectives

**DOI:** 10.3390/ijms21103528

**Published:** 2020-05-16

**Authors:** Giacomo Barchiesi, Marco Mazzotta, Eriseld Krasniqi, Laura Pizzuti, Daniele Marinelli, Elisabetta Capomolla, Domenico Sergi, Antonella Amodio, Clara Natoli, Teresa Gamucci, Enrico Vizza, Paolo Marchetti, Claudio Botti, Giuseppe Sanguineti, Gennaro Ciliberto, Maddalena Barba, Patrizia Vici

**Affiliations:** 1 UOC Oncologia, Ospedale dell’Angelo, 30174 Mestre, Italy; giacomo.barchiesi88@gmail.com; 2 Division of Medical Oncology 2, IRCCS Regina Elena National Cancer Institute, 00144 Rome, Italy; eriseld.krasniqi@ifo.gov.it (E.K.); laura.pizzuti@ifo.gov.it (L.P.); elisabetta.capomolla@ifo.gov.it (E.C.); domenico.sergi@ifo.gov.it (D.S.); antonella.amodio@ifo.gov.it (A.A.); patrizia.vici@ifo.gov.it (P.V.); 3 Department of Clinical and Molecular Medicine, Sant’Andrea Hospital, Sapienza University; Medical Oncology Unit, 00189 Rome, Italy; danielemarinelli@uniroma1.it (D.M.); paolo.marchetti@uniroma1.it (P.M.); 4 Department of Medical, Oral & Biotechnological Sciences, University G. D’Annunzio, 66100 Chieti-Pescara, Italy; natoli@unich.it; 5 Medical Oncology, Sandro Pertini Hospital, 00157 Rome, Italy; t.gamucci@libero.it; 6 Department of Oncological Surgery, Gynecologic Oncologic Unit, “Regina Elena” National Cancer Institute, 00144 Rome, Italy; enrico.vizza@ifo.gov.it; 7 Medical Oncology Unit B, Policlinico Umberto I, Sapienza University, 00161 Rome, Italy; 8 Department of Surgery, IRCCS Regina Elena National Cancer Institute, 00144 Rome, Italy; claudio.botti@ifo.gov.it; 9 Department of Radiation Oncology, IRCCS Regina Elena National Cancer Institute, 00144 Rome, Italy; giuseppe.sanguineti@ifo.gov.it; 10 Scientific Direction, IRCCS Regina Elena National Cancer Institute, 00144 Rome, Italy; gennaro.ciliberto@ifo.gov.it

**Keywords:** neoadjuvant treatments, breast cancer, endocrine therapy, hormonal therapy

## Abstract

In locally advanced (LA) breast cancer (BC), neoadjuvant treatments have led to major achievements, which hold particular relevance in HER2-positive and triple-negative BC. Conversely, their role in hormone receptor positive (HR+), hormone epidermal growth factor 2 negative (HER2-) BC is still under debate, mainly due to the generally low rates of pathological complete response (pCR) and lower accuracy of pCR as predictors of long-term outcomes in this patient subset. While administration of neoadjuvant chemotherapy (NCT) in LA, HR+, HER2- BC patients is widely used in clinical practice, neoadjuvant endocrine therapy (NET) still retains an unfulfilled potential in the management of these subgroups, particularly in elderly and unfit patients. In addition, NET has gained a central role as a platform to test new drugs and predictive biomarkers in previously untreated patients. We herein present historical data regarding Tamoxifen and/or Aromatase Inhibitors and a debate on recent evidence regarding agents such as CDK4/6 and PI3K/mTOR inhibitors in the neoadjuvant setting. We also discuss key issues concerning the optimal treatment length, appropriate comparisons with NCT efficacy and use of NET in premenopausal patients.

## 1. Introduction

Breast cancer is a heterogenous disease. Such heterogeneity is built on multifactorial ground, whose complexity is genetically rooted and reflects on phenotypic features [1]. Luminal cancers are characterized by the expression of estrogen receptors (ER) and/or progesterone receptors (PgR), while human epidermal growth factor 2 (HER2)-enriched tumors lack ER and PgR and show overexpression of the HER2 and/or amplification of the inherent gene. In triple-negative (TN) breast cancers, none of the prior targets are identifiable or adequately represented to be exploited for therapeutic purposes. This latter distinction into subgroups is of key importance due to the relevant differences in terms of prognosis and therapeutic weapons in current use [2,3].

The vast majority of breast cancers are diagnosed at an early stage, with about 5–15% of patients presenting with metastatic disease upfront. Neoadjuvant chemotherapy (NCT) is now a standard of care in locally advanced breast cancer. Initially, NCT was administered to increase the rate of breast conservative surgery (BCS) in patients with locally advanced breast cancer candidates for mastectomy [4]. Subsequent studies have shown that the achievement of pathologic complete response (pCR), defined as ypT0 ypN0, also correlates with favorable long-term clinical outcomes [5,6]. NCT confers the highest advantages in HER2 positive and TN breast cancer patients, for whom pCR can be obtained in 50–60% [7,8,9] of cases, while the inherent rates are lower in luminal cancer, where pCR is achieved on average in 10–20% of patients [6,7,8,9]. Indeed, ER-positive tumors are generally considered to be less sensitive to chemotherapy with respect to other subtypes [10]. In this subset of patients, a reasonable alternative or integrating strategy to cytotoxic chemotherapy can be represented by neoadjuvant endocrine therapy (NET).

NET has been evaluated in clinical trials for a long time [11]. In patients receiving NET, proven efficacy is coupled with high tolerability. This generally translates into excellent patients’ compliance, particularly, though not exclusively, in frail and elderly patients. In terms of health-related economics, endocrine agents are available at contained costs. The cost–efficacy balance is even more favorable when considering the easy delivery, relatively lower frequency access to health care facilities and limited efforts in terms of full time equivalent of health care providers, particularly when compared to NCT. [12]. However, NET it is not routinely used in clinical practice [13]. Nonetheless, in recent years, there has been a renewed interest in NET, mainly due to an increasingly high number of trials testing new drugs in combination with endocrine agents in ER-positive breast cancer patients. We herein present the available data on NET and critically discuss the most relevant findings. Hints on possible future scenarios are also provided.

## 2. Hormonal Agents

### 2.1. Tamoxifen

Tamoxifen was the first hormonal agent to be used in a clinical trial as a neoadjuvant hormonal agent. The encouraging evidence from pilot studies of tamoxifen used as an alternative to surgery in elderly, frail patients [11,14] led to prospective randomized clinical trial designed to evaluate the role of tamoxifen vs (vs) surgery in old patients, whose characteristics are recapitulated in Table 1, Section A. The trial from Robertson and colleagues enrolled 137 patients with operable hormone receptor positive (HR+) breast cancer, who received tamoxifen 40 mg per day or surgery. After 6 months of therapy, 55% of patients who had received tamoxifen responded to therapy. Even if the local relapse was higher in the tamoxifen arm (44% vs. 22%), no difference in OS was detected [15]. Similar results were obtained in another trial which enrolled 200 patients aged >70 years: local relapse was higher in patients treated with tamoxifen (20 mg per day). However, survival was not statistically different at a 6 year follow up [16]. Based on these findings, tamoxifen alone was then compared to surgery + tamoxifen. The most relevant trial which addressed the question on whether tamoxifen could provide a reasonable alternative to surgery was the GRETA trial, a phase III trial comparing tamoxifen with surgery + tamoxifen. Four-hundred seventy-four patients were enrolled to receive tamoxifen 20 mg (160 mg on day 1) or surgery and adjuvant tamoxifen 20 mg. The results showed that patients in the tamoxifen arm had 41.2% of clinical response (9.2% of complete responses and 33% of partial response). The authors observed 27 local progressions in the intervention group and 106 in the tamoxifen-alone group (*p* = 0.0001). In the surgery plus tamoxifen group, no differences were observed in overall survival (OS) by extent of surgery. Minimal surgery followed by tamoxifen was suggested as the most appropriate treatment in older patients [17]. 

### 2.2. Aromatase Inhibitors

The breakthrough of the third-generation aromatase inhibitors (AI), i.e., letrozole, anastrozole, and exemestane, as a preferred option for ER+ postmenopausal metastatic breast cancer patients [26,27] raised the question of whether the benefits observed in the advanced setting could be translated in early disease, thus providing an alternative to tamoxifen. The main features of trials of neoadjuvant AI are reported in Table 1, section B. A pilot study enrolled 24 postmenopausal patients to receive letrozole 2.5 mg, or 10 mg as NET. After three months of therapy, all the patients in the 2.5 mg arm and nine in the 10 mg arm had a response to therapy (five complete responses and 16 partial responses). Notably, 15 patients that were originally a candidate for mastectomy achieved a sufficient response to treatment, which allowed BCS [18].

A further study evaluated the efficacy of anastrozole as preoperative treatment in patients with ER+ breast cancer. Twenty-four patients received anastrozole 1 or 10 mg for three months before surgery. Most of patients were diagnosed with cT2 or cT3 tumors. The results showed a median tumor reduction volume of 85% in patients treated with anastrozole 1 mg. Of the 17 patients that were a candidate for mastectomy, 15 were suitable for BCS at the end of treatment [19].

Results from these studies invited subsequent trials comparing neoadjuvant AI vs. tamoxifen, whose characteristics are reported in Table 1, section C. 

The P024 trial compared letrozole 2.5 mg with tamoxifen 20 mg in 337 postmenopausal women with ER+/PR+ breast cancer. The primary endpoint was objective response to treatment. All the patients enrolled were not suitable for BCS, while 14% were not susceptible to surgery at diagnosis. Results showed significantly superior OR in the letrozole arm (55% vs. 36% *p* < 0.001). As a consequence, a higher percentage of patients in the letrozole arm had a BCS at the end of treatment (45% vs 35% *p* = 0.02) [20].

Another trial compared letrozole and anastrozole at different dosages to tamoxifen in ER+ postmenopausal women. The sample size was smaller than in the P024 (*n* = 71). However, some interesting conclusions could be drawn: letrozole and anastrozole showed similar efficacy, achieving a better clinical response compared to tamoxifen based on the caliper assessment, ultrasound and mammography; immunohistochemical analysis made on biopsies and surgery, showed a significant decrease in Ki-67 staining in patients treated with letrozole and anastrozole [21].

The efficacy of anastrozole was tested in the PROACT and IMPACT trials. The PROACT trial is a phase III trial, which compared 12 weeks of anastrozole 1 mg to tamoxifen 20 mg as NET in ER+ breast cancer. It is noteworthy that 20% of these patients were Japanese and chemotherapy could be administered concurrently to endocrine therapy. The primary endpoint was objective response (ObR). In patients treated exclusively with NET, anastrozole was superior to tamoxifen (ObR, 36.6% vs 24.2% at ultrasound evaluation *p* = 0.03; 48.6% vs. 35.8% at clinical evaluation *p* = 0.04), while in the overall population, even if the ObR was higher in the anastrozole group, the difference was not significant [22].

The IMPACT trial was a phase III study, which compared anastrozole 1 mg, tamoxifen 20 mg or the combination as a NET for 12 weeks [23]. The primary endpoint of the study was ObR. The IMPACT trial had also a secondary aim: testing if neoadjuvant treatments may ideally provide surrogate endpoints able to predict clinical outcomes in the adjuvant setting. By study design, the IMPACT trial was equivalent to the ATAC trial, a phase III study investigating the efficacy of anastrozole in the adjuvant setting [28]. Results showed that there was no difference in terms of ObR between the three arms (anastrozole, 37%; tamoxifen, 36%; and the combination, 39%, *p* = 0.61). Conversely, among patients requiring mastectomy at study entrance (*n*:124 out of 330 patients enrolled), the percentage of patients allocated to neoadjuvant treatment who resulted eligible to BCS following NEC was significantly higher in patients treated with anastrozole than in those allocated to tamoxifen (46% vs. 22%). The resulting odds ratio (OR) and 95% confidence interval (CI) were as it follows: OR 2.94; 95%CI 1.11–7.81; *p* = 0.03). Unfortunately, the trial failed to predict surrogate endpoints in the adjuvant setting. Indeed, in the ATAC trial, anastrozole showed superiority to tamoxifen in terms of disease-free survival (DFS). 

Exemestane was also tested as neoadjuvant treatment of 42 postmenopausal ER+ patients with locally advanced breast cancer. Exemestane was administered in monotherapy for 16 weeks. Overall, BCS was delivered to the 73.7% and 57.1% of the patients in the exemestane and control group, respectively [29]. Semiglazov et al. conducted a randomized clinical trial comparing 3 months of exemestane or tamoxifen in 151 patients as a preoperative treatment [24]. The primary endpoint was ObR. More patients in the exemestane arm had a response to treatment (exemestane 76% vs. tamoxifen 40%; *p* = 0.05) and more often underwent BCS (exemestane 37% vs. tamoxifen 20%; *p* = 0.05) compared to the tamoxifen group.

Based on the results obtained, which demonstrated better outcomes in terms of ObR and BCS for the AI compared to tamoxifen, a specific trial, the ACOSOG Z1031, addressed the question on whether there was a specific AI superior to the others. In this phase II trial, 377 ER+ (Allred score 6–8) breast cancer patients were randomized to anastrozole, exemestane or letrozole as preoperative treatment. The primary endpoint was clinical response. Results showed that there were no differences in terms of surgical outcomes and ki-67 reduction between the three arms. Substantially, the three drugs were biologically equivalent [30]. 

## 3. Menopausal Status

The vast majority of trials of hormonal agents in the neoadjuvant setting enrolled postmenopausal patients. In the IMPACT, PROACT, P024, and ACOSOGZ1031 trials, postmenopausal status was an inclusion criterion. Indeed, limited data are available in premenopausal patients. To our knowledge, a single-phase III trial evaluated 197 premenopausal ER+ HER2 negative breast cancer patients in Japan to receive anastrazole or tamoxifen plus goserelin for 24 weeks before surgery. The primary endpoint was best overall tumour response assessed with calliper, which was significantly higher in the anastrozole arm compared to tamoxifen (70.4% vs. 50.5% at callipers evaluation *p* = 0.003; 58.2% vs. 42.4% at ultrasound *p* = 0.027) and more patients in the anastrozole arm received BCS (86% vs. 69%) [25]. 

## 4. Duration of Treatment

The experience gained from clinical trials of neoadjuvant cytotoxic chemotherapy suggests the appropriate window for NET to fall into a 3 to 6 months range, but a precise duration has not been established yet. In clinical practice, a time window of 3 to 4 months of therapy is routinely offered to these patients, although some data suggest that this period could be insufficient. 

Several trials have explored a longer duration (>6 months) of NET aiming at finding the optimal extent of treatment and the limit of maximum response. A phase IIb trial enrolled 32 patients to receive letrozole for 4–8 months before surgery. All the patients were considered not suitable for BCS at diagnosis, due to disease extension. The primary endpoint was tumor regression and BCS eligibility. Results showed that the majority of patients achieved a partial response or complete response during the first 4 months. However, prolonging NET resulted in a further benefit in terms of tumor volume reduction: 67% vs. 64% at 8 and 4 months, respectively *p* = 0.05 [31]. Another trial enrolled 182 patients to neoadjuvant letrozole for 3 months or longer. Among them, 63 took letrozole for more than 3 months, 38 received it for more than 12 months and 23 for more than 24 months. The main reason for prolonging letrozole was an insufficient response to therapy for allowing a BCS. The results showed a continuous reduction in median clinical tumor volume in patients who took letrozole for more than 3 months [32]. More recently, a prospective randomized study enrolled 120 patients to receive 4 months (arm A), 8 months (arm B) or 12 months (arm C) of letrozole before surgery. All the patients were postmenopausal and unfit for chemotherapy. The primary endpoint was clinical response. Results showed that the majority of responses (partial + complete) were observed in patients who took letrozole for 12 months (Overall response rate, ORR, was 95%, 86.8% and 45% in arms C, B and A, respectively). This translated into a higher rate of BCS for patients in arm C, compared to B and A, although at a not statistically significant extent [33]. A further trial addressing the optimum NET length to achieve disease downstaging and allow for BCS showed a median duration of treatment was 7.5 months [34].

## 5. Hormone Therapy vs. Chemotherapy

As previously stated, ER+ HER2 negative breast cancer tumors are considered less sensitive to chemotherapy if compared to other subtypes, such as triple negative and HER2 positive tumors. Little data are available regarding a direct comparison between cytotoxic therapy and hormonal agents in the neoadjuvant setting: a phase II trial enrolled 239 postmenopausal patients to receive anthracycline- and taxane-based chemotherapy or neoadjuvant endocrine therapy with exemestane or anastrozole for 3 months. The primary endpoint was ORR. No significant differences emerged between the groups compared. However, there was a suggestion for higher rates of BCS in patients having received an aromatase inhibitor (33% vs. 24%; *p* = 0.06). The duration of neoadjuvant chemotherapy, i.e., 3 months, which does not represent the standard of care, may have at least partly obscured NET efficacy and contributed to minimized differences across the trial arms [35].

The GEICAM/2006-03 trial is another phase II trial which enrolled 95 patients to receive chemotherapy or exemestane preoperatively [36]. Premenopausal patients were eligible. Patients were randomized to receive four cycles of epirubicin-cyclophosphamide (EC) followed by four cycles of docetaxel or exemestane (plus goserelin in premenopausal patients) for 24 weeks. The primary endpoint was clinical response assessed by breast magnetic resonance. Overall, results showed that clinical response was somewhat more favorable in patients treated with chemotherapy compared to patients treated with exemestane (66% vs. 48%; *p* = 0.08). This difference gained full statistical significance in premenopausal women (*n* = 24) (75% vs. 44%, *p* = 0.03).

A systematic review and meta-analysis including 20 studies and 3490 patients showed no significant differences between AI endocrine therapy and neoadjuvant chemotherapy, in terms of clinical response rate (*p* = 0.85), radiological response rate (*p* = 0.12) and BCS rate (*p* = 0.07; at the price of a more severe toxicity in patients treated with neoadjuvant chemotherapy (NCT) [37]. More recently, a retrospective analysis has evaluated 140 patients who received NCT or NET. Patients received endocrine therapy until maximum clinical response. Approximately, 50% of patients in the chemotherapy arm received a combination of antracyclines, taxanes and platinum. Results showed no differences in terms of response to treatment. The median length follow-up was 4 years. No differences emerged in terms of locoregional recurrences (*p* = 0.87) and distant recurrences (*p* = 0.98) across the two arms [38].

Another trial investigated retrospectively the efficacy of NET or NCT in locally advanced lobular breast cancer, a breast cancer subtype which accounts for 10–15% of breast cancer and is often characterized by a higher expression of hormonal receptors, low grading and lower ki67 values if compared to ductal carcinoma [39]. A pilot study with letrozole as neoadjuvant treatment showed good clinical outcomes in patients with lobular cancer (76% of median tumor volume reduction measured clinically and 73% of median tumor volume reduction assessed by ultrasound after 3 months of therapy) [40].

Thus far, the largest retrospective study evaluated approximately 6000 patients, of whom 855 received NET and 5087 received NCT. When patients’ characteristics were compared, those who were treated with NET were older, with more co-morbidities and smaller tumors. Adjusted ten-year OS showed no difference in relevant outcomes across the study arms [41].

## 6. Molecular Alterations in Early Luminal Breast Cancer

In ER+ early breast cancer (eBC), endocrine therapy has considerably reduced breast cancer recurrence and mortality [42]. Still, up to 20% of patients diagnosed with eBC will eventually relapse, thus becoming metastatic. The vast majority of endocrine resistant tumors are not the consequence of ER loss, which occurs in approximately 10% of cases [43]. More often, endocrine resistance results from ER reactivation. This process can be caused by the crosstalk between ER with other grow factor receptors and/or the onset of gain-of-function mutations. In addition, novel investigational approaches, both in the pre- and clinical settings, have considerably added to the current knowledge of endocrine resistance. In more detail, molecular profiling of patients enrolled in neoadjuvant or peri-operative trials has contributed interesting clues in terms of underlying pathogenetic mechanisms and target identification/validation. In a recent work published by Giltnane et al., the authors evaluated 143 patients receiving letrozole for two weeks before surgery [44]. Genomic profiling on patients’ tumors revealed changes in gene expression and ER gene fusions associated with resistance to therapy. Among the alterations most commonly associated with endocrine resistance in ER+ eBC, the onset of mutations in the PI3K pathway, including PIK3CA, PTEN and AKT1 among its main actors, deserves mentioning [45]. In preclinical models, an aberrant activation of PI3K pathway was related to an acquired resistance to ER depletion [46]. However, clinical evidence from the clinical setting seems not to show worse outcomes in eBC patients harboring PI3K alterations [47]. PTEN and AKT1 mutations are more frequent in advanced ER+ cancer [48]. In this setting, the addition of drugs which specifically target this pathway to ET, such as Alpelisib (a PI3KCA inhibitor) or Capivasertinib (an AKT inhibitor) has improved patients’ outcomes [49,50]. Another common mechanism of resistance to ET is represented by alterations in receptor tyrosine kinases. Among them, ERBB2 has long been associated with reduced sensitivity to antiestrogen treatment [51]. Recent findings showed that in non-HER2-amplified patients, HER2-activating mutations were associated with endocrine resistance [52]. In addition, fibroblast grow factor receptor 1 (FGFR1) alterations have been also described in association with endocrine resistance. More specifically, FGFR1 amplification occurs in 10–15% of eBC [53]. A recent analysis of more than 1000 patients diagnosed with eBC showed that luminal A cancer expressing FGFR1 had a shorter DFS compared to negative patients (HR 3.341; *p* = 0.008) [54]. Possible mechanisms of resistance can rely also on deficiencies in DNA-repair genes. A high mutational load is associated with poor prognosis in ER+ breast cancer [55]. In a seminal work by Sansone et al., molecular profiling of patients undergoing NET with letrozole showed that estrogen deprivation induced a metabolically quiescent state where the ER expression was terminated [56]. More recently, there has been a renewed interest regarding the interaction between ER+ breast cancer and immune system/inflammatory response, probably on the wave of the relevant goals achieved in treatment of other malignancies including metastatic lung cancer, melanoma and TNBC [57,58,59]. Historically, ER+ tumors have been considered immunogenically “cold” cancers because of low TILs and PD-L1 expression [60,61]. More recently, the discovery that ERalfa downregulates PD-L1 expression has raised the hypothesis that an anti-estrogen treatment could positively affect PD-L1 levels [62].

## 7. Current Status and Future Applications of NET

We already discussed the clinical benefits of NET and its main possible advantages. However, the hormonal approach in the neoadjuvant setting has also provided additional data that have recently renewed interest in this field. Recent trials have been increasingly focused on the identification of reliable predictive/prognostic markers of efficacy/resistance. 

Data from the P024 trial database were used to generate the preoperative endocrine prognostic index (PEPI) score, which combined biomarker-related and pathological data. In more detail, the PEPI score included pathological tumor stage, nodal involvement, ER expression, and Ki67 percent expression (%), which all demonstrated an independent prognostic role for recurrence and death after recurrence. The PEPI score was validated using data from the IMPACT trial [63], with more favorable outcomes being shown in patients with T1 tumors and a PEPI score of 0, i.e., with a Ki67% less than 2.7% and ER expression. 

The P024 trial, IMPACT trial and ACOSOGZ0131 showed that a reduction in Ki67 values after 16 weeks, 2 weeks and 2–4 weeks, respectively, correlated with better relapse free survival (RFS) in multivariate analysis. Ki67 measured at 2–4 weeks of treatment is now the most accepted surrogate marker for NET and its drop is often used as clinical endpoint in randomized trials, with a Ki67 value >10% following treatment being usually considered as the cut-off point to identify non-responder patients. That threshold has been proposed based on results from the IMPACT trial and POL trial [64] which showed that a Ki67 value >10% after 1 month of therapy was associated with a higher PEPI score (*p* = 0.01) and a worse RFS (*p* = 0.0016) [65].

The use of the PEPI score and Ki67 as predictive biomarkers is still partly shadowed by limits which may at least partly impair their effective reliability. First, tumor biopsies remove only a small part of neoplastic mass and may thus be poorly representative of the actual tumoral cell population. On this basis, a still debated issue relates to considering the mean Ki67 value from multiple bioptic samples instead of one single Ki67 value. Further possible limitations stem from the introduction in clinical practice of serially profiled biopsies in the course of treatment, which would increase patients’ management costs and may generate or add to patients’ discomfort.

Additional trials have addressed the outcomes of endocrine therapy in a peri-operative window, along with the role of biomarkers in informing therapeutic decisions in the adjuvant setting. The “Perioperative Endocrine Therapy_ Individualizing Care (POETIC)” phase III trial randomly allocated 4486 postmenopausal ER+ HER2- breast cancer patients to peri-operative aromatase inhibitor (POAI) for 14 days before and 14 days after surgery vs. no peri-operative treatment. This trial failed in meeting its primary aim, since no significant differences emerged in terms of time of recurrence (TTR) between the groups compared (*p*:0.37) [66]. Data on the added value of Ki67 evaluation at 2 weeks from surgery (Ki672w), that is, at the end of the peri-operative window of endocrine treatment, were supportive. A Ki672w equal to or greater than 10 was significantly associated with a higher risk of 5-year relapse (*p* ≤ 0.001). In these patients, evidence from the POETIC trial suggests therapeutic choices beyond the standard of care [66]. Validating a biomarker-driven strategy for treatment of post-menopausal women with ER+ HER2- invasive breast cancer is also the aim of the ALTERNATE trial [67]. In this phase III randomized clinical trial, postmenopausal women with a cT2–4 N0-3 M0 ER+ HER2- invasive breast cancer were randomized to (I) neoadjuvant anastrozole followed by surgery, and then anastrozole for 4.5 years; (II) neoadjuvant fulvestrant, surgery, followed by fulvestrant for the first 18 months and anastrozole for 3 years; and (III) the combination of anastrozole and fulvestrant. The biomarker-driven treatment strategy is based on the assessment of Ki67 at 4 and 12 weeks of NET and evaluation of the preoperative endocrine prognostic index (PEPI score). For women with tumor Ki67 > 10% at 4 weeks (mandatory) or 12 weeks (optional) of NET, the switch to neo-adjuvant chemotherapy was recommended. Similarly, women having completed 6 months of NET and with pT3/4 or pN1–3 or Ki67 > 2.7% residual disease at surgery were recommended to receive adjuvant chemotherapy. Genomics holds great promise in the identification and validation of predictive/prognostic biomarkers in breast cancer patients undergoing NET. A four-gene classifier of clinical response established based on the level of two genes associated with immune signaling, i.e., IL6ST and NGFRAP1, and the levels of two proliferation genes, i.e., ASPM, MCM4, was tested in 89 postmenopausal patients treated with neoadjuvant AIs. Changes in this signature in matched 2-week on-treatment biopsies, i.e., in primary breast tissue samples collected through biopsies performed before treatment and after 2 weeks, showed greater predictive power than pretreatment biopsies alone. This signature showed also predictivity on recurrence-free survival and breast-cancer-specific survival (*p* = 0.029 and 009, respectively). Validation was performed in a blinded, completely independent set of patients treated with anastrozole in the anastrozole-only-arm of a neo-adjuvant trial [68]. Furthermore, the ACOSOGZ0131 trial used the PAM50 algorithm for an intrinsic subtype assignment (LumA, LumB, normal, HER2 enriched and basal-like). The RNA-based analysis, available for about two thirds of the population, seems useful for excluding uncommon non-luminal intrinsically endocrine-therapy-resistant tumors and confirm that the Ki67 value is a good surrogate for the genomic molecular classification [30]. A suggestion for a potential future application of a PAM50 profiling-based approach which may further help explain tumor heterogeneity and its impact on treatment outcome in the neoadjuvant setting may come from a recently published study. The authors built genome metrics by applying R programming to the PAM50 profiles of 674 luminal A breast cancers from the METABRIC cohort. Results were validated in an independent set of 509 luminal A cases from the TCGA. Two metrics were defined and cases were assigned to only two groups, i.e., the “Pure” and “Admixed luminal A“ groups, which were compared by clinical and molecular features in reference to survival outcomes. These latter were significantly less favorable for cases allocated to the admixed group. Hints for future directions and use of similar approach in studies of endocrine therapy in the neo-adjuvant setting may include combining more homogeneous, well clinically annotated cohorts to assess specific types of admixture and exploiting methods to use whole transcriptome data and integrative genomics to measure admixture with greater accuracy [69]. More recently, our group has published a retrospective analysis of 59 elderly patients treated with NET. In this relatively limited, though well characterized cohort, biomarkers of DNA damage and repair, i.e., the phosphorylated ataxia-teleangectasia and Rad3-related protein (pATR), phosphorylated ataxia-telangiectasia mutated (ATM) kinase, and phosphorylated H2A Histone Family Member X (γ-H2AX), were evaluated against Ki67 changes. Patients staining positive for γ-H2AX and pATM at baseline had the greatest likelihood of achieving a decrease in Ki67 values and increase in ∆γ-H2AX in paired tissue samples. These biomarkers showed prognostic relevance, since associated with more favorable outcomes in terms of event-free survival and OS [70].

Neoadjuvant endocrine therapy has been widely used in clinical trials as a platform to test new drugs and to design adjuvant studies exploiting neoadjuvant data. Adding experimental drugs to neoadjuvant endocrine treatment has important benefits: it allows a complete pharmacodynamic and pharmacokinetic biomarker evaluation of the tested drugs, thus increasing the knowledge and developmental stage of the molecules and helping to understand resistance mechanisms.

Combination therapies have been evaluated for years in neoadjuvant trials, with the first results being quite disappointing. AIs were associated with several agents but none of them resulted in improved clinical outcomes [71,72,73]. The breakthrough of CDK 4/6 inhibitors (palbociclib, ribociclib, abemaciclib), an innovative class of agents approved for first and second line in the metastatic setting in combination with aromatase inhibitors or fulvestrant, based on a PFS and OS improvement in phase III trials [74,75,76], has renewed interest in this field. The characteristics of trials of combination therapy with AIs plus novel therapeutics are summarized in Table 2. To our knowledge, five clinical trials have been published [77,78,79,80,81,82] to date. Even if study design, primary endpoints and number of patients differ from study to study, preliminary results showed some analogies: the rate of Ki67 response and complete cell cycle arrest (CCCA), defined as a Ki67 < 2.7%, seems higher when adopting the combination strategy compared to ET alone. The NeoPalAna study [77] is a phase II trial which evaluated anastrozole monotherapy vs. anastrozole + palbociclib in 50 patients with clinical stage II or III, ER+ HER2 negative breast cancer. Enrolment of premenopausal patients was allowed. CCCA at C1D15 of therapy was the primary endpoint of the trial. Eligible patients received 4-week anastrozole monotherapy and were then randomized to continue anastrozole or receive anastrozole + palbociclib. Adding palbociclib to anastrozole improved CCCA rate at C1D15 when compared to anastrozole monotherapy at C1D1 (87% vs. 26%; *p* = 0.001). Ki67 levels were reduced from baseline to C1D1 by anastrozole and from C1D1 to C1D15 by palbociclib addition (*p* = 0.01). In the NEOMONARCH 2 trial, 223 postmenopausal ER+ HER2 negative with clinical stage I-III breast cancer patients were randomized to receive anastrozole monotherapy, abemaciclib monotherapy or their combination. The primary endpoint of the trial was Ki67 reduction after 2 weeks of therapy. More patients in the combination arm achieved a Ki67 reduction compared to the monotherapy arm (92.62% vs. 63.24%; *p* < 0.001) [78].

Modifications of Ki67 were also the primary endpoint of the PALLET [54] and MONALEESA-1 trial [79,81]. The PALLET trial is a phase II trial which compared letrozole monotherapy (arm A) to letrozole + palbociclib administered to patients in different modalities (letrozole for 2 weeks, then palbociclib plus letrozole to 14 weeks, arm B; palbociclib for 2 weeks, then palbociclib plus letrozole to 14 weeks, arm C; palbociclib plus letrozole for 14 weeks, arm D). The MONALEESA-1 trial is a pre-surgical study which compared letrozole monotherapy vs. the combination of letrozole and ribociclib 400 or 600 mg. In both trials, combination therapy led to a greater Ki67 reduction, compared to the monotherapy arms (96% and 92% for letrozole + ribociclib 400 mg and 600 mg arm, respectively, vs. 69% for monotherapy arm in the MONALEESA-1 trial; median log-fold change in Ki-67 was greater with palbociclib plus letrozole compared with letrozole in PALLET trial). In PALLET trial patients in the combination arm achieved also a greater CCCA rate (90% vs. 59%; *p* < 0.001) [79,81].

Besides the anti-proliferative effect analysis, clinical response to treatment was also evaluated: the available data from trials demonstrated a radiological response which ranged between 40% and 55%, in line with previous clinical trials employing aromatase inhibitor monotherapy. One of the major clinical issues of CDK4/6 plus AI combination concern pCR achievement. pCR was obtained in 3.7% of patients in the NEOMONARCH II and in one patient out of 10 enrolled in N007 [80], while in NEOPalAna no pathological complete responses were reported, and in PALLET trial no differences were observed between the experimental arms and control arm. Therefore, whether the apparent better antiproliferative effect of aromatase inhibitor combined with CDK4/6 inhibitors translates into a clinical benefit is still under debate. Several neo/adjuvant trials are addressing the question [82,85,86].

Probably the most relevant challenge of combining endocrine therapies with CDK 4/6 inhibitors remains the direct comparison with chemotherapy. Very few data are available, since only one study has been published: the NEOPAL trial. In this trial, 106 patients with Prosigna luminal B or luminal A and node positive breast cancer were randomised to receive letrozole and palbociclib for 19 weeks or chemotherapy (fluoruracil, epirubicin, cyclophosphamide for three cycles followed by docetaxel for three cycles). The primary endpoint was residual cancer burden (RCB). RCB 0-I was observed in 7.7% of letrozole-palbociclib arm vs. 15.7% of chemotherapy arm. pCR was more common in the NCT arm (5.9% vs. 3.7%). However, clinical responses and BCS were similar between the two groups (75% and 69%, respectively). The safety profile favored the letrozole plus palbociclib arm compared to NCT (2 vs. 17 serious adverse events were recorded) [87].

The combination strategy aimed at enhancing the effectiveness of NET also includes inhibitors of PI3K–AKT–mTOR pathway. Deregulation of PI3K–AKT–mTOR pathway is a well-known mechanism of primary or secondary endocrine resistance [88]. More specifically, PI3KCA mutations occur in approximately 35% of breast cancer and are more common in ER+ tumors [89]. Several agents targeting this pathway have been tested, e.g., everolimus, an mTOR inhibitor, and alpelisib, a PI3KCA inhibitor, showing a clinical benefit in advanced disease [49,90]. Everolimus has also been evaluated in the neoadjuvant setting; a phase II trial compared 4 months of letrozole plus placebo or letrozole plus everolimus in 270 postmenopausal breast cancer patients. The primary endpoint was clinical response by palpation. Results showed that clinical responses were higher in letrozole plus everolimus arm (68.1% vs. 59.1 *p* = 0.062) [83]. More recently, the PI3KCA inhibitor taselisib has been investigated in combination with letrozole as neoadjuvant therapy. Results from the LORELEI trial showed that adding taselisib to letrozole before surgery significantly improved the clinical outcomes of ER+ HER2− breast cancer patients. The trial enrolled 334 patients, having as co-primary endpoints ORR assessed by magnetic resonance and pCR rate. Patients in the taselisib arm had a better ORR compared to the placebo arm (50% vs. 39.3%, odds ratio [OR] 1.55, 95% CI 1.00–2.38, *p* = 0.049), but there was no significant difference between the groups for pCR. Taselisib showed greater activity among the 152 patients who had PIK3CA mutant cancer cells detected at baseline, with 56.2% showing an ORR compared to 38% of patients who received placebo ([OR] 2.03, 95%CI 1.06–3.88, *p* = 0.033) [84].

## 8. Window of Opportunity Trials

Since being placed within a pre-surgical timeframe, window of opportunity trials (WoTs) deserve mention within the context of neo-adjuvant trials. In WoTs, the intervention of interest is confined to the time interval elapsing between breast biopsy and breast surgery. The availability of serially collected tissue samples allows for evaluating target modulation and/or the biomarkers most commonly tested for their prognostic/predictive relevance. Ki67 is a validated biomarker widely used in WoTs as a surrogate of long-term outcomes. On average and based on prior findings, treatment administration is programmed on the short length, more commonly of about 2 weeks. Indeed, short-term Ki67changes in the course of neoadjuvant therapy of primary breast cancer with anastrozolo, tamoxifene or their combination were detectable at 2 and 12 weeks from treatment initiation and correlated with recurrence-free survival [91]. However, more recent evidence from a trial of 44 hormone receptor positive breast cancer patients has shown significantly reduced Ki67 values following a 7-day course of pre-surgical tamoxifen [92]. 

By nature, WoTs conduct may fuel ethical concerns. Indeed, substantial evidence of safety is required in the absence of a short-term therapeutic advantage, particularly in patients who are potentially curable exclusively by surgery. However, WoTs potential in terms of general applicability of findings concerning outcome prediction, future trial design and avoidance of ineffective treatment remain virtually limitless [93].

## 9. Ongoing NET Trials

The critical role played by cyclin-dependent kinases in regulating cell cycle transitions, along with the achievements from the metastatic setting in terms of PFS and OS, has increasingly fueled interest in the use of CDK4/6 inhibitors in the neoadjuvant setting [94]. At the time of writing, we identified 12 ongoing trials of CDK4/6 inhibitors combined with hormonal agents in ER+ breast cancer patients that were candidates for NET. The related characteristics are summarized in Table 3 (Table 3a–c). Some of these trials deserve a more in-depth description. The antitumor immune response mediated by the programmed death 1/programmed death ligand 1 (PD1/PD-L1) and cytotoxic T lymphocyte antigen-4 inhibitors provides a proof-of-principle for immunotherapy use in breast cancer [95]. In the NCT03573648, NCT04075604, and NCT04088032, the combined use of CDK4/6 inhibitors and immunotherapic agents is foreseen. In more detail, in the phase II trial NCT03573648 trial, 40 patients are being randomly allocated to tamoxifen +/− palbociclib and then newly undergoing breast biopsy, MRI, and blood draw. The anti–PD-L1 human monoclonal antibody Avelumab will be added to both arms. Patients will be treated for three cycles of avelumab with tamoxifen +/− palbociclib. Patients completing all four cycles of therapy will then undergo MRI and surgery [96]. In the phase II randomized multi-arm trial NCT04075604, 136 ER+/HER2− breast cancer patients will be evaluated with reference to safety and efficacy endpoints following the administration of palbociclib and anastrozole with or without nivolumab, a human-programmed death receptor-1 (PD-1) blocking antibody [97]. 

In the early phase I NCT04088032 trial, 10 patients with locally advanced ER+ HER2- breast cancer will be treated with Abemaciclib, AI, and the programmed death-ligand 1 (PD-L1) inhibitor Durvalumab (MEDI4736). The primary hypothesis relates to safety issues, while the secondary hypothesis relates to an increase in stromal tumor-infiltrating lymphocytes (TILs) following NET administration in combination with abemaciclib and durvalumab for four cycles (16 weeks) [107]. 

Of further notice, the use of neoadjuvant CDK4/6 inhibitors combined with AI in ER+ HER2+ breast cancer patients, who represent the elective population target for the phase II trial NCT02907918. The recruitment target is forty-eight patients with stage II-III ER+ HER2+ breast cancer for treatment assignment according to a single-arm study design [101].

The use of multigene prognostic tests as screening tools characterizes both the NCT03628066 and NCT02400567 trials. In the NCT03628066, the recurrence score (RS) obtained through Oncotype DX is functional to patients’ stratification into two cohorts of approximately 38 patients each. The two cohorts will include patients with an RS less than 11 and between 11 and 26, respectively. Patients from both cohorts will be identically treated for the first 6 weeks, i.e., will receive letrozole, palbociclib, and goserelin. At week 6, patients from both cohorts with a Ki67 less than 10% level on (repeated) core biopsy will continue receiving prior therapy for six cycles. Conversely, patients whose cancer shows Ki67 values greater than or equal to 10% will discontinue study therapy and either undergo surgery or initiate NACT. In this trial, stratification of patients by RS will thus allow outweighing the impact of genomic features delineated by multigene prognostic testing against the primary and secondary outcomes in two patient cohorts undergoing the same treatment [100]. In the NCT02400567 phase II trial, 125 postmenopausal women with stage II-IIIA breast cancer will be randomized to either neoadjuvant CT or NET based on letrozole combined with palbociclib. The investigators propose integrating innovative diagnostic approaches such as the PAM50 signature and the residual cancer burden (RCB) tumor response. Thus, in this trial, randomization will minimize selection bias, while the results from PAM50 screening will help to control for the influence of genomic features on treatment outcomes [102].

## 10. Conclusions

The body of knowledge on neoadjuvant endocrine therapy has dramatically grown in recent years. From a clinical standpoint, NET represents a feasible and effective treatment option, especially in ER+ HER2 negative postmenopausal patients, with AI administered preoperatively for 3–6 months being the gold standard. A promising therapeutic strategy is represented by the combination of an AI and a target agent such as CDK4/6 inhibitors or PI3K–AKT–mTOR pathway inhibitors, although further studies are needed to confirm preliminary data.

However, some inherent key issues remain unaddressed. The optimal length of treatment has yet to be established, along with the efficacy of NET in premenopausal patients and its efficacy based on the direct comparison of NET with NCT. Beyond the clinical aspects, NET has now a wide range of applications in research, as it provides a versatile framework for testing new therapeutic agents, identifying and validating prognostic and predictive biomarkers, and clarifying the mechanisms of endocrine resistance. 

## 11. Materials and Methods

We performed a literature search of three major databases (DBs), i.e., MEDLINE, EMBASE and the Cochrane Central Register of Controlled Trials (CENTRAL). These DBs were last accessed on the 31st October 2019. To the purposes of the work herein presented, we combined both text words and Medical Subject Headings (MeSH) thesaurus related to the following terms: neoadjuvant endocrine therapy, “neoadjuvant hormone” and “breast cancer”, “letrozole” and “neoadjuvant”, “tamoxifen” and “neoadjuvant”, Anastrozole and “neoadjuvant”, pathologic complete response, survival outcomes. Proceedings and abstracts related to selected annual scientific events that occurred during the last ten years were also considered, including the San Antonio annual breast cancer symposium, the American Society of Clinical Oncology (ASCO) and the American Association of Cancer Research (AACR) annual meetings. For each reference judged eligible by at least one of the authors involved in the search and screening process, we consulted the reference list and used the “related features” tool made available in PubMed to identify the adjunctive literature of potential interest. Studies were considered for inclusion if enrolling patients with locally advanced BC patients, defined as non-metastatic and eligible for neoadjuvant treatment and subsequent definitive surgery. 

## Figures and Tables

**Table 1:** Clinical trials of tamoxifen vs. surgery (section A); Aromatase inhibitors’ monotherapy (Section B) and aromatase inhibitors vs. tamoxifen (section C).

Sections	First Author and Reference Number	Study Population	*n*	Treatment and Length (M)	BCS (%)	Endpoint/s* & Assessment Method (AM)	F. up (M)
Section A: tam vs surgery	Robertson JFR et al. [15]	Operable Elderly (>70 y) ER+ Bca	137	Tam 40 mg vs. Surgery Lenght: 6	NA^1^	LR: 44 vs 22% AM: NR OS: 89.4 vs 84.7%, *p* > 0.05	25
	Gazet JC et al. [16]	Operable Elderly (>70 y) ER+ Bca	200	Tam 20 mg vs. Surgery Lenght: NA	NA^2^	LR: 53 vs 36% DR or mixed: 8 vs 14% AM: NR OS: 67 vs 72%, *p* > 0.05; DFI: 39 vs 50%, *p* > 0.05	72
	Mustacchi G et al. [17]	Operable Elderly (>70 y) ER+ Bca	474	Tam 20 mg vs. Surgery→Tam Length: 60	NA^3^	LR: 45.2 vs 11.2%, *p* < 0.0001 AM:Mx and Clinical examination BCD: 23.8 vs 23%, *p* = 0.18	80
Section B: AI	Dixon JM et al. [18]	Operable Post-menopausal ER+ Bca	24	Letrozole 2.5 mg vs. Letrozole 10 mg Length 3	100%^4^	Reduction of Tumor Size AM: 1. Calliper a. Letrozolo 2.5 mg: CR: 5/12 PR: 7/12 SD: 0/12 b. Letrozolo 10 mg CR: 0/12 PR: 9/12 SD: 3/12 2. US&Mx a. Letrozolo 2.5 mg CR: 1/12 PR: 9/12 SD: 2/12 b. Letrozole 10 mg CR: 0/12 PR: 8/12 SD: 4/12	NR
	Dixon JM et al. [19]	Operable Post-menopausal ER+ Bca	24	Anastrozole 1 mg vs. Anastrozole 10 mg Lenght: 3	88.2^5^	Reduction of Tumor Size AM: Calliper: 89.3 vs 99.6% Imaging: 1.US: 80.5 vs 69.6% 2.Mx: 73.6 vs 69.6%	NR
Section C: AI vs Tam	P024 trial [20]	Inoperable Post-menopausal ER+ Bca	337	Letrozole 2.5 mg vs. Tam 20 mg Lenght: 4 M	45 vs 35%, *p* = 0.02	OR AM: Clinical examination: 55 vs 36%, *p* < 0.001	NR
	Miller WR et al. [21]	Post-menopausal ER+ Bca	71	Letrozole (A) vs. Anastrozole (B) vs. Tam (C) Lenght: 4	NR^6^	OR AM: US <25%: 8% A, 17% B, 2% C; 25–50%: 17% A, 9% B, 52% C; >50%: 75% A, 78% B, 46% C	NR
	Cataliotti L et al. [22]	Post-menopausal ER+ Bca	451	Anastrozole 1 mg vs. Tam 20 mg Lenght: 3	43 vs 31%, *p* = 0.04	OR AM: US 39 vs 35%, *p* = 0.29	NR
	Smith IE et al. [23]	Post-menopausal ER+ Bca	330	Anastrozole 1 mg (A) vs. Tam 20 mg (B) vs. Anastrozole 1 mg + Tam 20 mg (C) Lenght: 4	44 vs 31%, *p* = 0.23	OR AM: Calliper: %RC + RP and SD in: A: 0.34 and 0.42 B: 0.33 and 0.51 C: 0.37 and 0.43 US: %RC + RP and SD in: A: 0.21 and 0.26 B: 0.19 and 0.31 C: 0.26 and 0.26	NR
	Semiglazov V. et al. [24]	Post-menopausal ER+ Bca	151	Exemestane 25 mg vs. Tam 20 mg Length: 3	36.8 vs 20%, *p* = 0.05	OR AM: Clinical examination: 76 vs 40%, *p* = 0.05	NR
	Masuda N et al. [25]	Pre-menopausal ER+ Bca	197	Anastrozole 1 mg + Goserelin vs Tam 20 mg + Goserelin Length: 6 M	86 vs 68%	OR AM: Calliper: 70.4 vs 50.5%, *p* = 0.003	NR

* Endpoint/s other than rate of breast conservative surgery. ^1,2,3^ NA: Not applicable. Rate of breast cancer surgery is not an endpoint in these studies. ^4^ “Letrozole used in a neoadjuvant setting is highly effective, producing clinically beneficial reductions in tumor volume allowing all patients to have breast conserving surgery” quoting citation from the abstract conclusion. ^5^ “Of the 17 patients who would have required a mastectomy at initiation of treatment, 15 were suitable for breast conservation after anastrozole treatment” quoting citation from the abstract conclusion. ^6^ BCS rate is not clearly reported. A 91% may be assumed based on the abstract. Quoting citation “Results showed that in these selected groups of patients a reduction in tumor volume with treatment was observed in 43 of 47 cases (91%).” *n*: Number of study participants; BCS: Breast conservative surgery; AI: Aromatase inhibitors; F. up: median follow up in months; Bca: Breast cancer; BCD: Breast cancer deaths; BCS: Breast conservative surgery; DFI: Disease free interval; ER: estrogen receptor; AM: Assessment Method; OR: Objective response; OS: Overall survival; Tam: Tamoxifen; M: months; NR: Not reported; y: years; mg: milligrams; LR: Local Recurrence; DR or Mixed: Distant Recurrence or mixed, this latter including both local and distant recurrence; US: Ultrasound; Mx: Mammography.

**Table 2:** Combination therapy with NSAI plus novel therapeutics in the neoadjuvant setting. Included patients were diagnosed with ER+, HER2- stage I-II-III BC.

Study	Design	Drugs	Primary Endpoint	Patients (*n*)	Main Results	pCR
NeoPalAna [77]	Phase II, single arm	A → A + Pal	CCCA at C1D15	50	CCCA rate significantly higher after adding Pal to A (C1D15 87% vs. C1D1 26%, *p* < 0.001)	none
NeoMONARCH 2 [78]	Phase II, three arms	A vs. Abe vs. A + Abe	change in Ki-67 (C1D1 - C1D15)	223	In A + Ac Ki67 reduction was higher compared to A (92.6% vs. 63.24%; *p* < 0.001)	3.7% in A + Abe arm
PALLET [79]	Phase II, four arms	L vs. L + Pal in different schedules	change in Ki-67 (C1D1 - C1D15), CR in 14 weeks	307	Ki67 reduction significantly higher in L + Pal. CR not different.	3.3% in L + Pal arms
N007 [80]	Prospective, single arm	L + Pal	CR and PEPI	20	17 pts showed clinical response	5%
MONALEESA-1 [81]	Phase II, three arms	L vs. L + Rib in different doses	change in Ki-67 (C1D1 - C1D15)	14	Ki67 reduction higher in L + Rib	NA
NEOPAL [82]	Phase II, two arms	L + Pal vs. CHT	RCB	106	RCB higher in CHT arm	3.8% in L + Pal 5.9% in CHT
Everolimus & Letrozole [83]	Phase II, two arms	L + plc vs. L + Eve	CR by palpation	270	CR higher in L + Eve arm (68.1% vs. 59.1%, *p* = 0.06)	1.4% in L + Eve 0.8% in L
LORELEI [84]	Phase II, two arms	L + plc vs. L + Tas	ORR (by MRI) and pCR	334	ORR 50% in L + T vs. 39% in L + Plc, *p* = 0.049	1.8% in L + Tas 0.6% in L + plc

ER+: estrogen receptor positive; HER2-: human epidermal growth factor receptor 2 negative; BC: breast cancer; *n*: number; pCR: pathologic complete response; A: anastrozole; Pal: palbociclib; CCCA: complete cell cycle arrest (central Ki67 ≤ 2.7%); C1D1: cycle 1 day 1; C1D15: cycle 1 day 15; Abe: abemaciclib; L: letrozole; CR: clinical response; PEPI: preoperative endocrine prognostic index; Rib: Ribociclib; NA: not available; CHT: chemotherapy; RCB: residual cancer burden; plc: placebo; Eve: everolimus; Tas: Taselisib; ORR: overall response rate; MRI: Magnetic Resonance Imaging.

**Table 3:** a: ongoing trial with Palbociclib in the neoadjuvant setting. b: ongoing trials with Ribociclib in the neoadjuvant setting. c: Ongoing trials with Abemaciclib in the neoadjuvant setting.

Section	Study	Design	Population	Arms	Primary Endpoint	Secondary Endpoints	Status
a	NCT02764541 [98]	Phase II WoT	Lobular Bca	WoT: 2w Letrozole or Tamoxifen A:Letrozole B:Letrozole +Palbociclib	(A) Ki67 changes^1^ (B) pCR rate	-ORR -DFS -OS	Recruiting
	NCT03969121 [99]	Phase III	ER+ pre/perimenopausal Bca	A: AI + Palbociclib + OFS; B: AI+ OFS	-PEPI score -EPclinscore	-cCR -Ki67 change -pCR -BCS rate	Recruiting
	NCT03573648 [96]	Pilot trial	ER+ Bca	A: Tamoxifen+ Avelumab+ Palbociclib B: Tamoxifen+ Avelumab	-CCR by MRI	-Safety and tolerability	Recruiting
	NCT03628066 [100]	Phase II	ER+ Premenopausal; Oncotype Dx at baseline Low/Intermediate RS required	Arm A: RS 1-11 Letrozole+ Palbociclib+ OFS Arm B: RS 12-26 Letrozole+ Palbociclib+ OFS	-Complete cell cycle arrest	-ORR -pCR Imact of Oncotype DX on cCR and pCR	Recruiting
	NCT04075604 [97]	Phase II	ER+/HER2- Breast Cancer	Arm A: Anastrazole + Palbociclib+ Nivolumab; Arm B: Anastrozole +Palbocilib→ Palbociclib +Nivolumab; Arm C: Anastrazole + Palbociclib	-Dose limiting toxicity -RCB	-Safety -pCR -ORR -BCS rate	Recruiting
	NCT02907918 [101]	Phase II	ER+ HER2 + pre/post- menopausal; patients	Arm A: Letrozole+ Palbociclib+ Trastuzumab +/− OFS	-pCR	-Safety -QoL	Recruiting
	NCT02400567 [102]	Phase II	PAM50 low/intermediate risk	Arm A: FECx3→Txt ×3; Arm B: Letrozole+ Palbociclib	-RCB	-Response assessed clinically and by US -Safety -BCS rate	Active, not recruiting
	NCT04137640 [103]	Phase IV	LABC; Luminal A	Arm A: Letrozole+ Palbociclib Arm B: ECx4q28→Txt × 4q28	-clinical response	-BCS -Ki67 expression -PEPI -PFS -OS	Not recruiting
b	NCT02712723 [104]	II	ER+ HER2-postmenopausal patients; clinical stage II-III	Arm A: Letrozole Arm B: Letrozole + Ribociclib 600 mg Arm C: Letrozole + Ribociclib 400 mg	-PEPI rate at surgery	Complete cell cycle arrest -pCR -cCR -RFS	Active, not recruiting
	NCT03283384 [105]	II	ER+ HER2-postmenopausal patients; clinical stage I-III	2w Letrozole, then: A) Ki67 <1% →Letrozole B) Ki67 ≥ 1% → Letrozole + Ribociclib C) Ki67 ≥ 1% → ACdd→T	-Complete cell cycle arrest	-pCR -AEs -EFS -OS	Recruiting
c	NCT04293393 [106]	II	Pre-postmenopausal HR+ HER2- patients; high-intermediate risk, clinical stage II-III breast cancer	Arm A: AC×4→T×4 Arm B: Letrozole + Abemaciclib +/− OFS	-RCB 0-I rate	-Changes in Ki67 -PEPI score 0-1 -Clinical response by -MRI -BCS rate -iEFS -AEs	Not yet recruiting
	NCT04088032 [107]	I, pilot study	postmenopausal HR+ HER2- patients; clinical stage II-III breast cancer	Arm A: AI + Abemaciclib + Durvalumab	-AEs	-pCR	Not yet recruiting

WoT: Window of opportunity trials; Bca: Breast cancer; BCS: breast conservative surgery; cCR: complete clinical response; DFS: disease free survival; EPclin: endopredict clinical score; MRI: magnetic resonance imaging; OFS: ovarian function suppression; ORR: overall response rate; OS: overall survival; PEPI: preoperative endocrine prognostic index; pCR: pathologic complete response; PFS: progression free survival; RS: Recurrence score; QoL: quality of life RCB: residual cancer burden; w: weeks; US: ultrasound; EC: epirubicin + cyclophosphamide; Txt: Docetaxel. AEs: adverse events; ER: estrogen receptor; cCR: complete clinical response; EFS: event free survival; OS: overall survival; PEPI: pre-operative endocrine prognostic index; RFS: recurrence free interval; ACdd: Anthracycline + cyclophosphamide dose dense; T: taxane. AC: Anthracycline-Cyclophosphamide; AEs: adverse events; AI: aromatase inhibitor; BCS: breast conservative surgery; iEFS: invasive event free survival; MRI: magnetic resonance imaging; OFS: ovarian function suppression; pCR: pathologic complete response; PEPI: pre-operative endocrine prognostic index. ^1^ Ki67 changes between baseline and breast biopsy performed at day 15.

## Abbreviations

LA	locally advanced
BC	breast cancer
HR+	hormone receptor positive
pCR	pathological complete response
NCT	neoadjuvant chemotherapy
NET	neoadjuvant endocrine therapy
ER	estrogen receptors
PgR	progesterone receptors
HER2-	hormone epidermal growth factor 2 negative
TN	triple negative
BCS	breast conservative surgery
ObR	objective response
PEPI	preoperative endocrine prognostic index
DBs	databases
CENTRAL	Cochrane Central Register of Controlled Trials
MeSH	Medical Subject Headings
ASCO	American Society of Clinical Oncology
AACR	American Association of Cancer Research
OS	overall survival
OR	odds ratio
CI	confidence interval
DFS	disease free survival
ORR	overall response rate
EC	epirubicin-cyclophosphamide
RFS	relapse free survival
pATR	phosphorylated ataxia-teleangectasia and Rad3-related protein
ATM	phosphorylated ataxia-telangiectasia mutated
γ-H2AX	phosphorylated H2A Histone Family Member X
CCCA	complete cell cycle arrest
RCB	residual cancer burden
eBC	early breast cancer
PI3K	phosphatidylinositol-4,5-bisphosphate 3-kinase
PIK3CA	phosphatidylinositol-4,5-bisphosphate 3-kinase catalytic subunit alpha
AKT1	RAC-alpha serine/threonine-protein kinase
PTEN	Phosphatase and tensin homolog
FGFR1	fibroblast grow factor receptor 1
TILs	Tumor-infiltrating lymphocytes
PD-L1	Programmed death-ligand 1
PD-1	Programmed death-1
POAI	peri-operative aromatase inhibitor
TTR	time to recurrence
WoTs	window of opportunity trials
CDK4/6	cyclin-dependent kinase 4 and 6
